# The Erythrocyte, a Novel Disease-Mediator for COVID-19 Vasculopathy?∗

**DOI:** 10.1016/j.jacbts.2022.01.012

**Published:** 2022-03-28

**Authors:** Carlo Brugnara

**Affiliations:** Department of Laboratory Medicine, Boston Children’s Hospital, and the Department of Pathology, Harvard Medical School, Boston, Massachusetts, USA

**Keywords:** arginase, COVID-19, endothelial dysfunction, reactive oxygen species, red blood cells

**Figure:**
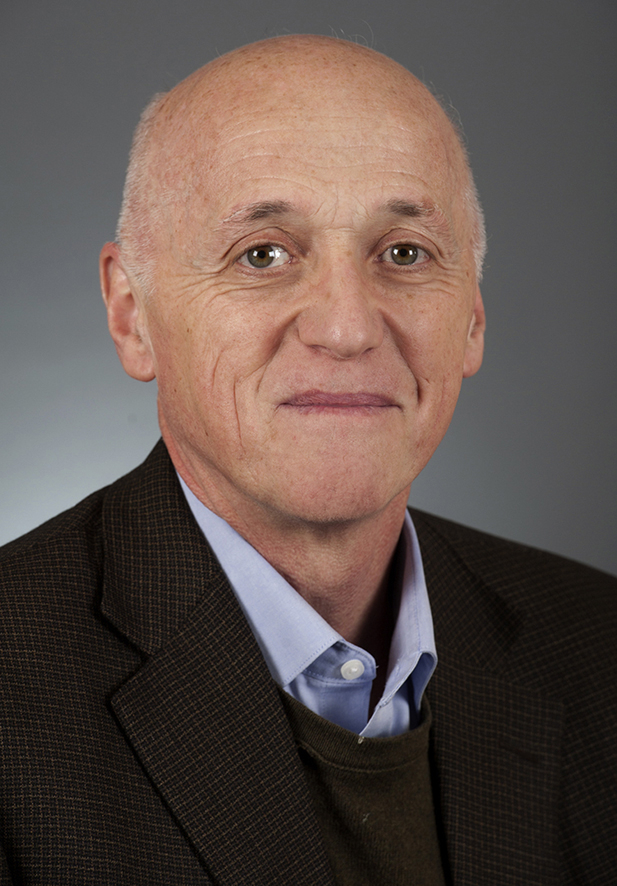

Over the last 25 years, we have learned that a novel function of human erythrocytes involves the regulation of nitric oxide (NO) delivery and availability to peripheral endothelium, a hypoxia-driven mechanism that plays a role in the control of vascular tone and blood pressure. The original concept of the erythrocyte being only a NO scavenger was challenged by several lines of evidence that suggested the presence of erythrocyte-mediated vasodilation in hypoxic conditions, driven, at least in part, from a net NO generation by erythrocytes and possibly adenosine triphosphate release from hypoxic erythrocytes. Several mechanisms have been proposed for NO generation: formation of S-nitrosohemoglobin (at the Cys-93 residue of the hemoglobin β subunit) and/or reduction of nitrite to NO or dinitrogen trioxide; and enzymatic production of NO within the erythrocyte by an endothelial NO synthase (eNOS). We have learned that both endothelium and erythrocytes are capable of generating NO via their own eNOS. eNOS functional activity is greater in endothelial cells, but the greater number of erythrocytes may compensate for this difference and render these 2 pathways similar in overall magnitude. How much each of these 2 NO producing pathways might contribute to local control of vascular tone was not known until recent work in a mouse model. This model showed that both erythrocyte-derived and endothelium-derived NO are key in regulation of vascular tone, and that they behave as 2 functionally independent separate arms.^1^ Although endothelial cell eNOS is the prevalent regulatory mechanism, the contribution of erythrocyte-derived NO seems to be key in maintaining normal vascular resistance.^1^ These erythrocyte-centered NO regulatory mechanisms are relevant not only for normal physiology but also in hematological diseases. Intravascular hemolysis of sickle erythrocytes leads to release of oxy-hemoglobin into plasma, which not only promotes reactive oxygen species (ROS) formation but also has exceptionally high NO scavenging capabilities. This leads to a NO depletion state that impairs NO-dependent vasodilation.^2^ Thus, NO deprivation is a key component of the endothelial dysfunction and inflammatory vasculopathy that accompanies hemolysis and release of hemoglobin and heme in the circulation of patients with sickle cell anemia.

In this issue of *JACC: Basic to Translational Science*, thanks to the work by Mahdi et al^3^ from Karolinska Institutet, Stockholm, Sweden, we now learn that human red cells are affected in their function by COVID-19. Red cells may be important players in the vasculopathy associated with COVID-19, which is a key mediator of the organ failure and thrombotic complications of the disease. Although the initial focus of COVID-19−related research has been on thrombotic mechanisms, recent attention has been paid to erythrocytes as possible important mediators of COVID-19 disease pathophysiology at the vascular level. Key findings of the work by Mahdi et al are: 1) the reactive hyperemia index was significantly reduced in patients with COVID-19 both during the acute phase and at follow-up; 2) endothelium-dependent and independent relaxations were blunted by erythrocytes (but not plasma) from patients with COVID-19 compared with normal control erythrocytes. This was, at least in part, mediated by increased arginase activity and ROS generation and possibly by diminished NO export from COVID-19 erythrocytes. Measurements of elevated interferon-γ in red cell lysates of patients with COVID-19 suggested a possible cytokine-mediated triggering mechanism; 3) ROS generation was increased in erythrocytes of patients with COVID-19; and 4) erythrocyte-mediated abnormalities were no longer present in patients who were followed 4 months after the acute event, with persistence of the in vivo blunted reactive hyperemia index.

The evidence of vascular dysfunction is convincing, and the persistence 4 months after the acute illness is remarkable, especially in a vascular bed that is not among the COVID-19 targets. Equally tantalizing is the evidence for functional abnormalities and increased ROS generation of erythrocytes collected during the acute phase of the infection and their return to normal after 1 life span (120 days). This reflects the generation of a new normal cohort of cells, as expected. The increased arginase activity and ROS generation are likely to lead to a NO-restricted state, an important mediator of vasculopathy, as studies in sickle cell disease have demonstrated.

The suggestion of Mahdi et al^3^ that erythrocytes are important mediators of vascular dysfunction during the acute phase of COVID-19 is reasonable and worth investigating further, but some limitations of this hypothesis need to be pointed out. Although the in vitro data are generally supportive of the hypothesis, the in vivo data suggest otherwise, showing essentially the same impairment of the reactive hyperemia index in the acute and follow-up phases, with an apparent normality of erythrocyte behavior at follow-up. If erythrocytes play an important role, one would have expected an amelioration of the in vivo reactive hyperemia index impairment when erythrocyte functions are normalized. An additional concern is that all patients were on various levels of oxygen therapy, which makes it impossible to discern COVID-19−specific alteration versus oxygen-induced alteration of the red cell membrane and function. It is known that oxygen therapy promotes oxidation of membrane lipids, as shown by increased hydroperoxide values in the erythrocyte membrane of preterm infants exposed to oxygen therapy,^4^ and that erythrocyte viscosity and aggregation are increased in patients with COVID-19 who require oxygen supplementation.^5^ The changes in erythrocyte viscosity and aggregation are largely mediated by increased fibrinogen, suggesting that they may be secondary to hypercoagulability and vascular changes associated with pulmonary lesions.^5^ Studies in patients with type 1 diabetes mellitus and who were exposed to hyperbaric oxygen showed decreased iNOS activity and/or expression in lymphocytes and increased plasma arginase activity,^6^ which suggested that a NO-restricted state could be a consequence of oxygen therapy.

In conclusion, this is a well done and provocative study, which suggests the need for additional in-depth mechanistic studies to determine the role of erythrocyte abnormalities in the vasculopathy associated with COVID-19.

## Funding Support and Author Disclosure

The author has reported that he has no relationships relevant to the contents of this paper to disclose.
